# Simple and Highly Discriminatory VNTR-Based Multiplex PCR for Tracing Sources of *Aspergillus flavus* Isolates

**DOI:** 10.1371/journal.pone.0044204

**Published:** 2012-09-17

**Authors:** Dong Ying Wang, Leila Hadj-Henni, Simon Thierry, Pascal Arné, René Chermette, Françoise Botterel, Inès Hadrich, Fattouma Makni, Ali Ayadi, Stéphane Ranque, Wei Yi Huang, Jacques Guillot

**Affiliations:** 1 Parasitology Department, College of Animal Science and Technology, Guangxi University, Nanning, China; 2 ANSES, Laboratoire de Santé Animale, UMR BIPAR, Maisons-Alfort, France; 3 ENVA, Laboratoire de Santé Animale, UMR BIPAR, Maisons-Alfort, France; 4 UPE, Laboratoire de Santé Animale, UMR BIPAR, Créteil, France; 5 Laboratoire de Biologie Moléculaire Parasitaire et Fongique, Faculté de Médecine de Sfax, Sfax, Tunisia; 6 Aix-Marseille Université, UMR MD3, Marseille, France; 7 APHM, Timone, Laboratoire de Parasitologie-Mycologie, Marseille, France; Soonchunhyang University, Republic of Korea

## Abstract

*Aspergillus flavus* is second only to *A. fumigatus* in causing invasive aspergillosis and it is the major agent responsible for fungal sinusitis, keratitis and endophthalmitis in many countries in the Middle East, Africa and Southeast Asia. Despite the growing challenge due to *A. flavus*, data on the molecular epidemiology of this fungus remain scarce. The objective of the present study was to develop a new typing method based on the detection of VNTR (Variable number tandem repeat) markers. Eight VNTR markers located on 6 different chromosomes (1, 2, 3, 5, 7 and 8) of *A. flavus* were selected, combined by pairs for multiplex amplifications and tested on 30 unrelated isolates and six reference strains. The Simpson index for individual markers ranged from 0.398 to 0.818. A combined loci index calculated with all the markers yielded an index of 0.998. The MLVA (Multiple Locus VNTR Analysis) technique proved to be specific and reproducible. In a second time, a total of 55 isolates from Chinese avian farms and from a Tunisian hospital have been evaluated. One major cluster of genotypes could be defined by using the graphing algorithm termed Minimum Spanning Tree. This cluster comprised most of the isolates collected in an avian farm in southern China. The MLVA technique should be considered as an excellent and cost-effective typing method that could be used in many laboratories without the need for sophisticated equipment.

## Introduction

Members of the genus *Aspergillus* are ubiquitous filamentous fungi widely distributed in the environment. About 250 species have been described but only a few (about 20) have been reported to cause opportunistic infections in humans and animals. The most important pathogens in this genus are *Aspergillus fumigatus, Aspergillus flavus, Aspergillus niger, Aspergillus terreus,* and *Emericella nidulans* (anamorph: *Aspergillus nidulans*) [1]. *Aspergillus* fungi release large amounts of airborne asexual spores (conidia). As a result of its ubiquitous presence, people and animals are constantly exposed to *Aspergillus* conidia, which can lead to colonisation, allergic manifestations or invasive infection depending on host immunity. Birds are considered to be much more susceptible than mammals [2]. Respiratory aspergillosis remains a major cause of morbidity and mortality in turkey poults in large confinement houses, quails, marine birds that are brought into rehabilitation, captive raptors, and penguins being maintained in zoological parks [3], [4]. In Europe and North America, the majority of human and animal aspergillosis are caused by *Aspergillus fumigatus* [1], [5], [6]. The second most frequent pathogenic species is *A. flavus* [7], [8]. In many countries from the Middle East, Africa and Southeast Asia however, *A. flavus* is the principal *Aspergillus* species causing human invasive forms of aspergillosis but also sinusitis, keratitis, endophthalmitis and cutaneous infections [9]–[12]. In a recent epidemiological survey in avian farms in the South of China (Guangxi Province), we demonstrated that *A. flavus* was the major airborne filamentous fungus isolated from air, eggshells and the respiratory tract of birds. The species *A. flavus* is also known to cause environmental aflatoxin contamination, particularly in maize and peanuts, which leads to substantial economic loss worldwide [13].

Tracking the sources of contamination is essential to prevent *A. flavus* infection in humans or animals. For that purpose, molecular tools have been proposed [14] but the number of techniques validated for *A. flavus* is much lower than that currently used for *A. fumigatus* in hospitals or animal facilities.

The Multiple Locus Variable-number tandem-repeat Analysis (MLVA) is based on polymorphism of tandemly repeated genomic sequences called VNTR (Variable-Number Tandem-Repeats). VNTRs are classically separated into microsatellites (up to 8 bp) and minisatellites (9 bp and more) [15]. The MLVA technique has been used for the genotyping of many bacterial pathogens. It allows resolving closely related isolates for the investigation of disease outbreaks and provides information on the phylogenetic patterns among isolates [16]–[18]. The usefulness of MLVA was recently demonstrated for *A. fumigatus* [19]. The MLVA technique can be performed with simple electrophoretic equipment and the judicious association of several VNTR primers in a single reaction tube may considerably reduce the number of PCRs required for a single isolate.

The objective of the present study was to develop a new typing method based on the detection of VNTRs in the filamentous fungus, *A. flavus*.

## Materials and Methods

### Origin of Fungal Isolates

In order to develop a scheme for MLVA and select discriminant VNTR markers, a total number of 36 *A. flavus* geographically or temporally unrelated isolates were selected. Thirty isolates were collected from the environment, from the pharynx or the respiratory tract of birds or from human cases of aspergillosis. Six reference strains (UMIP20.65, UMIP954.67, UMIP597.69, UMIP855.64, UMIP1145.76 and NRRL3357) were also included. To test the MLVA technique, a second group of 55 isolates was examined. These isolates represented distinct epidemiological situations: (i) 41 isolates from avian farms in Southern China and (ii) 14 isolates from human cases of invasive aspergillosis in Tunisia.

*Aspergillus* isolates were microscopically identified after cultivation on Malt Agar plates at 37°C until conidia formation. For all isolates, the species identification was confirmed by amplification and sequencing of partial *ß-tubulin* gene using primer set ßtub1 and ßtub2 [20], [21].

### DNA Isolation

For each isolate, conidia were collected from the pure culture and transferred into a microtube for extraction. DNA extraction was performed with QIAamp DNA miniKit (Qiagen) according to the manufacturor’s instructions.

### Selection of VNTR Markers

The availability of sequences of *A. flavus* (strain NRRL3357) (http://www.aspergillusflavus.org/) allowed us to identify exhaustively tandem-repeat sequences using the Tandem Repeat Finder on-line software (http://tandem.bu.edu/trf/trf.html) [22]. Loci with tandem repeats consisting of more than 20 nucleotides and more than 3 repeats were selected. Primers were designed using Primer Express® 2.0 software (Applied Biosystems). PCR were performed in a total volume of 15 µl containing 1–5 ng of DNA, 1x mixed buffer and 0.5 µM of each primer. The initial denaturation step at 95°C for 10 min was followed by 35 cycles consisting of denaturation at 95°C for 40 s, primer annealing at 61°C for 40 s, and elongation at 72°C for 40 s. The final extension step was at 72°C for 10 min. Six microliters of amplification product were loaded onto a 2% standard agarose gel. Gels stained with ethidium bromide were visualized under UV light, and photographed. The size marker used was a Quick-load 100-bp ladder (New England BioLabs, Ipswich UK).

Taking into account the maximum and minimum size of the repeats, combinations of VNTR markers were finally tested to obtain a multiplex technique with a clear and unambiguous separation of amplicons on agarose gels. All the combinations were tested by double, triple and fourfold PCR. The primer annealing temperature was the same (61°C) for all multiplex PCRs.

### Specificity

To test the specificity of the MLVA technique, isolates from other *Aspergillus* species were also included: 4 reference strains of the *Flavi* section (*A. parasiticus* UMIP1142.76, *A. tamarii* UMIP1017.70 and *A. oryzae* UMIP1042.72, UMIP1141.76), 3 strains of other sections of the genus *Aspergillus*: *A. fumigatus* (CBS 14489), *A. niger* (CBS 733.88) and *A. nidulans* (CBS 589.65).

### Stability and Reproducibility

The stability of the VNTR markers was estimated by analysis of 5 distinct isolates of *A. flavus* subcultured 12 times in 2 months.

The reproducibility of the method was assessed by the analysis of 25 isolates in 2 different units situated in two different buildings of the Animal Health Laboratory of ANSES (Agence Nationale de Sécurité Sanitaire) at Maisons-Alfort, France, and by 2 different technicians.

### Discriminatory Power

The discriminatory power was calculated by using the Simpson index of diversity [23].

### Clustering Analysis

Amplicon size was determined with Quantity One software package version 4.6.9 (Bio-Rad Laboratories, USA). The number of repeats in each allele was derived from the amplicon size. The size of flanking sequences was subtracted from the band size and the number was divided by the repeats size. The result of this calculation corresponded to the number of repeats. Data were analyzed with Bionumerics software package version 6.5 (Applied-Maths, Saint-Martens-Latem, Belgium) as a character dataset. Two different techniques were used to represent the relationships between isolates [18]: a phenogram using phenetic UPGMA method and a graphing algorithm termed Minimum Spanning Tree (MST). The priority rule for constructing MST was set in order that the type that had the highest number of single-locus variants (SLVs) would be linked first. A cutoff value of maximum differences of one VNTR out of 8 was applied to define cluster in the MST method.

## Results and Discussion

The use of the Tandem Repeat Finder software allowed the detection of 24 tandem-repeat loci (with a repeat unit larger than 20 bp) within the genome of *A. flavus*. Out of 24 tandem-repeat loci, 14 had a homology of more than 90% between the different repeats and a number of repeats higher than 3. Only 8 loci were finally deemed suitable for genotyping because they displayed variation (more than 3 alleles among the 36 tested isolates) and were present in all the isolates. Final markers were located on 6 different chromosomes (1, 2, 3, 5 7 and 8). Characteristics of final VNTRs and respective primer sets are listed in table 1. For multiple MLVA analysis, 4 pairs of markers were finally chosen: the combinations of AFL2-66 and AFL3-236, AFL1-75 and AFL5-66, AFL5-81 and AFL8-135, AFL7-57 and AFL7-78. These combinations allowed a clear and unambiguous separation of amplicons on agarose gels. Multiple (more than 2)-bands patterns were never detected.

**Table 1:** Characteristics of VNTR markers for fingerprinting of *Aspergillus flavus.*

VNTR markers	Primer sequences (5′ to 3′)	Unit repeat size (bp)	Range of repeat number	Simpson diversityindex*	Marker location (non coding region or name of the gene if coding)
AFL1-75	GTCAGAGTGCTGTTGGGCG CGTCTCCCAGGCCGTTAGT	75	0–8	0.494	Chromosome 1, non coding
AFL2-66	CGCGAATGTCGATGATCACT AACAGGTAGGGCTGGGTTCC	66	3–6	0.618	Chromosome 2, GPI anchored protein, putative
AFL3-236	CAGAATTTTCAGTTAGCAAAGTGCTC TAAGACTTGGAGATATGTGACAAGGCTATA	236	1–3	0.541	Chromosome 3, non coding
AFL5-66	TCCACAGGCTGTATCGTTATCCT CAGTGACCCTTTCGGTGAAGAC	66	0–5	0.740	Chromosome 5, conserved hypothetical protein
AFL5-81	GGTTGCATCACAGTTATAGCGCT CCGGCACGACTGTGGAC	81	2–9	0.802	Chromosome 5, conserved hypothetical protein
AFL7-57	CACCGCAATGGAGCACAAG TGGTCGAGCTGTTCCTGGA	57	6–17	0.818	Chromosome 7, hypothetical protein
AFL7-78	GCTTCGTCATTGGCCCAT CTCGATCAATGTGTACTCATAAATGCT	78	2–6	0.655	Chromosome 7, conserved hypothetical protein
AFL8-135	GGTTTGCAGTGAGGATCTGCT GATGTGAGCCAGGCCATTG	135	0–3	0.398	Chromosome 8, conserved hypothetical protein

* Each index was calculated with the results from the 36 unrelated *Aspergillus flavus* isolates including 6 reference strains.

When VNTR primer sets were tested with DNA from *A. fumigatus*, *A. niger* and *A. nidulans* no amplification was observed. On the contrary with DNA from *Aspergillus* species from the Flavi section, amplification was obtained with 4 to 5 out of 8 markers (AFL1-75, AFL2-66, AFL5-66 and AFL8-135 for *A. parasiticus* and *A. oryzae*; AFL1-75, AFL2-66, AFL3-236, AFL7-78 and AFL8-135 for *A. tamarii*). As a consequence, the observation of 8 amplicons following the combination of 8 VNTRs should be considered as specific of *A. flavus* and 3 markers (AFL1-75, AFL2-66 and AFL8-135) may be specific of the section *Flavi*.

The 35 samples (5 isolates subcultured 7 times in 2 months) used for the evaluation of stability were typed by MLVA and yielded exactly the same MLVA pattern for each isolate. The 50 samples used for the evaluation of reproducibility (25 isolates tested by 2 different technicians in 2 different laboratories) yielded exactly the same MLVA pattern.

Simpson diversity index was first calculated for each VNTR and for the panel of 8 markers tested on the 30 unrelated isolates and 6 reference strains. The index for individual markers ranged from 0.398 to 0.818. A combined loci index calculated with all of 8 markers yielded an index of 0.998.

A total number of 91 *A. flavus* isolates, including 6 reference strains were typed with the panel of 8 VNTRs. This analysis yielded 78 different genotypes, which corresponds to a combined loci index of 0.993. Among all genotypes, 71 were only found once. Five genotypes were shared by two isolates, one genotype was shared by three isolates and one genotype was shared by 7 isolates. Analysis of the details of those isolates in a single genotype revealed that 7 of them were isolated from eggshells in May and June 2010 in the same avian farm in China (farm A). Three isolates collected from eggshells and pharyngeal swabs (in laying hens) from the same farm shared a single but distinct genotype. Two isolates from another avian farm in China (farm B) shared a single genotype. The same situation was detected for two pairs of isolates collected in a hospital in Tunisia: one genotype corresponded to two isolates collected in bronchoalveolar lavages (from two different patients) and another one corresponded to two isolates collected from nasal swabs (from two distinct patients). More surprisingly, two unrelated isolates (one isolate from an avian farm in Nanning, China and the reference strain UMIP597.69) shared the same genotype. Although an identical genotype can occur by chance in unrelated isolates, a probable explanation for this result is that a contamination occurred during subculture.

**Figure 1:**
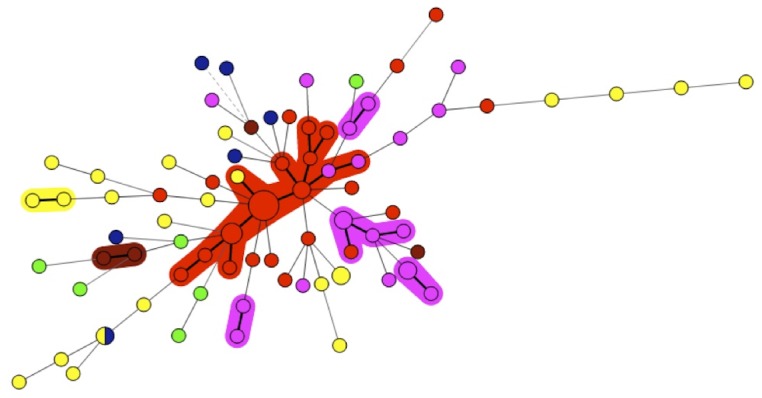
Minimum spanning tree of 91 *A. flavus* isolates based on categorical analysis of 8 VNTRs. Each circle represents a unique genotype. The diameter of each circle corresponds to the number of isolates with the same genotype. Genotypes connected by a shaded background differ by a maximum of one VNTR marker and could be considered a “clonal complex”. Thick connecting lines represent one marker difference; regular connecting lines represent two or three marker differences; thin interrupted lines represent four or more differences. The length of each branch is also proportional to the number of difference. Each epidemiological situation is represented by a specific colour: red for 32 isolates collected in an avian farm (A) in Guilin, China; yellow for 23 isolates collected other avian farms (B, C, D) in Nanning, China; purple for 20 isolates collected in patients and in the hospital of Sfax, Tunisia; green for 6 isolates collected from a stock of mouldy peanuts in Benin; brown for 4 isolates collected from the fur of domestic carnivores in France and blue for 6 reference strains.

UPGMA analysis did not allow a clear clustering of the isolates (data not shown) whereas the graphing algorithm termed Minimum Spanning Tree (MST) demonstrated one major cluster of isolates (figure 1). This cluster comprised 19 out of 32 isolates collected in the avian farm A in Guilin, China. Additional but smaller clusters could be defined for isolates collected in the hospital in Sfax or from avian farm B in China.

In a recent review, Hadrich *et al*. [14] listed the different molecular techniques, which have been tested so far for the typing of *A. flavus* or for the distinction between the clades or species within the *Flavi* section. Various DNA fingerprinting systems have been described such as restriction fragment length polymorphism (RFLP) [24]–[26], random amplified polymorphic DNA (RAPD) [27], [28], amplified fragment length polymorphism (AFLP) [29] and, very recently, microsatellite length polymorphism (MLP) [30]–[32]. Most of these typing techniques were developed in order to resolve closely related isolates for the purposes of outbreak investigation in hospitals and disease surveillance in humans. In only one study [31], the genetic markers (24 microsatellite loci and the mating type locus) were used to assess population structure and potential gene flow among *A. flavus* vegetative compatibility groups in sympatric populations in Arizona and Texas. The RAPD method was used for *A. flavus* probably because it requires simple equipment and no genomic sequence information, but it suffered from limited discriminatory power and reproducibility. MLP typing methods were proved to be highly discriminant and reproducible. Hadrich *et al*. selected 12 microsatellite markers for the typing of 63 *A. flavus* isolates (15 from Marseille, France and 48 from Sfax, Tunisia, including some of the isolates we examined in the present study) [30]. The use of all the markers yielded 35 different genotypes with a diversity index of 0.970. A 5 markers combination yielded 27 different genotypes with a diversity index of 0.952. In 2011, Rudramurthy *et al*. developed a multicolor microsatellite panel for genotyping of *A. flavus* [32]. Nine microsatellite markers were finally used for the typing of 162 clinical isolates from India. The diversity index for the individual markers ranged from 0.657 to 0.954. The diversity index of the panel of nine markers combined was 0.997. In order to compare MLVA and microsatellite typing techniques, we examined 20 *A. flavus* isolates from patients from Sfax hospital. This preliminary analysis indicated that the MLVA method yielded a larger number of genotypes than the microsatellite technique proposed by Hadrich *et al*. [30] (17 MLVA patterns versus 11 microsatellite profiles). Both techniques confirmed that iterative samplings on the same patients generally yielded to the isolation of different *A. flavus* genotypes. In further investigations a larger number of *A. flavus* isolates should be examined to obtain a precise comparison between MLVA and microsatellite typing techniques.

In conclusion, we developed a new molecular typing method for *A. flavus* based on the study of 8 VNTR markers. Size differences between alleles of the 8 selected VNTRs were large enough to allow a multiplex amplification and further efficient differentiating on agarose gel. This makes the present MLVA scheme easy to implement in laboratories with basic molecular biology equipment. The method showed a good reproducibility, which could be increased by the production of an internal ladder (including an example of each allele amplicon size). The MLVA was shown to be rapid and very discriminant. The VNTR patterns were incorporated in a specific database (http://minisatellites.u-psud.fr/MLVAnet/). On this website, it is now possible to compare *A. flavus* VNTR patterns using complete panel of 8 markers or just a selection of them. This database also allows to build dendrograms with the query [33].

## References

[1] Denning DW (1998) Invasive aspergillosis. Clin Infect Dis 26: 781–803. 9564455 10.1086/513943

[2] Tell LA (2005) Aspergillosis in mammals and birds: impact on veterinary medicine. Med Mycol Suppl 1, S71–73. 10.1080/13693780400020089 16110795

[3] Beernaert LA, Pasmans F, Van Waeyenberghe L, Haesebrouck F, Martel A (2010) *Aspergillus* infections in birds: a review. Avian Pathol 39: 325–331. 20954008 10.1080/03079457.2010.506210

[4] Arné P, Thierry S, Wang D, Deville M, Le Loc’h G, et al. (2011) *Aspergillus fumigatus* in poultry. Intern J Microbiol 746356. Epub 2011 Jun 14. 10.1155/2011/746356 PMC3150149 21826144

[5] Balajee SA, Kano R, Baddley JW, Moser SA, Marr KA, et al (2009) Molecular identification of *Aspergillus* species collected for the transplant-associated infection surveillance network. J Clin Microbiol 47: 3138–3141. 19675215 10.1128/JCM.01070-09 PMC2756904

[6] Lortholary O, Gangneux JP, Sitbon K, Lebeau B, de Monbrison F, et al (2011) Epidemiological trends in invasive aspergillosis in France: the SAIF network (2005–2007). Clin Microbiol Infect 17: 1882–1889. 21668573 10.1111/j.1469-0691.2011.03548.x

[7] Krishnan S, Manavathu EK, Chandrasekar PH (2009) *Aspergillus flavus*: an emerging non-*fumigatus Aspergillus* species of significance. Mycoses 52: 206–222. 19207851 10.1111/j.1439-0507.2008.01642.x

[8] Pasqualotto AC (2009) Differences in pathogenicity and clinical syndromes due to *Aspergillus fumigatus* and *Aspergillus flavus*. Med Mycol (Suppl 1): S261–270. 10.1080/13693780802247702 18654921

[9] Khairallah SH Byrne KA, Tabbara KF (1992) Fungal keratitis in Saudi Arabia. Doc Ophthalmol 79: 269–276. 1600844 10.1007/BF00158257

[10] Wong TY, Fong KS, Tan DT (1997) Clinical and microbial spectrum of fungal keratitis in Singapore: a 5-year retrospective study. Int Ophthalmol 21: 127–130. 9587828 10.1023/a:1026462631716

[11] Taj-Aldeen SJ, Hilal AA, Chong-Lopez A (2003) Allergic *Aspergillus flavus* rhinosinusitis: a case report from Qatar. Eur Arch Otorhinolaryngol 260: 331–335. 12883959 10.1007/s00405-002-0547-x

[12] Hedayati MT, Pasqualotto AC, Warn PA, Bowyer P, Denning DW (2007) *Aspergillus flavus*: human pathogen, allergen and mycotoxin producer. Microbiol 153: 1677–1692. 10.1099/mic.0.2007/007641-0 17526826

[13] Robens J, Cardwell FK (2003) The cost of mycotoxin managment to the USA: management of aflatoxin in the United States. J Toxicol 22: 139–152.

[14] Hadrich I, Makni F, Neji S, Cheikhrouhou F, Sellami H, et al (2011) A review molecular typing methods for *Aspergillus flavus* isolates. Mycopathol 172: 83–93. 10.1007/s11046-011-9406-x 21369748

[15] Vergnaud G, Denoeud F (2000) Minisatellites: mutability and genome architecture. Genome Res 10: 899–907. 10899139 10.1101/gr.10.7.899

[16] Schouls LM, van der Heide HG, Vauterin L, Vauterin P, Mooi FR (2004) Multiple locus variable-number tandem repeat analysis of Dutch *Bordetella pertussis* strains reveals rapid genetic changes with clonal expansion during the late 1990s. J Bacteriol 186: 5496–5505. 15292152 10.1128/JB.186.16.5496-5505.2004 PMC490882

[17] Top J, Schouls LM, Bonten MJM, Willems RJL (2004) Multiple-Locus Variable-Number Tandem Repeat Analysis, a novel typing scheme to study the genetic relatedness and epidemiology of *Enterococcus faecium* Isolates. J Clin Microbiol 42: 4503–4511. 15472301 10.1128/JCM.42.10.4503-4511.2004 PMC522339

[18] Wang YW, Watanabe H, Phung DC, Tung SK, Lee YS, et al (2009) Multilocus variable-number tandem repeat analysis for molecular typing and phylogenetic analysis of *Shigella flexneri* . BMC Microbiol 9: 278. 20042119 10.1186/1471-2180-9-278 PMC2806262

[19] Thierry S, Wang D, Arné P, Deville M, De Bruin B, et al (2010) Multiple-locus variable-number tandem repeat analysis for molecular typing of *Aspergillus fumigatus* . BMC Microbiol 10: 315. 21143842 10.1186/1471-2180-10-315 PMC3004892

[20] Balajee SA, Gribskov JL, Hanley E, Nickle D, Marr KA (2005) *Aspergillus lentulus* sp. nov., a new sibling species of *A. fumigatus* . Eukaryot Cell 2005 4: 625–632. 10.1128/EC.4.3.625-632.2005 PMC1087803 15755924

[21] Balajee SA, Nickle D, Varga J, Marr KA (2006) Molecular studies reveal frequent misidentification of *Aspergillus fumigatus* by morphotyping. Eukaryot Cell 5: 1705–1712. 17030996 10.1128/EC.00162-06 PMC1595351

[22] Benson G (1999) Tandem repeats finder: a program to analyze DNA sequences. Nuc Acids Res 27: 573–580. 10.1093/nar/27.2.573 PMC148217 9862982

[23] Hunter PR, Gaston MA (1988) Numerical index of the discriminatory ability of typing systems: an application of Simpson’s index of diversity. J Clin Microbiol 26: 2465–2466. 3069867 10.1128/jcm.26.11.2465-2466.1988 PMC266921

[24] Moody SF, Tyler BM (1990) Restriction enzyme analysis of mitochondrial DNA of the *Aspergillus* flavus group: *A. flavus*, *A. parasiticus*, and *A. nomius* . Appl Environ Microbiol 56: 2441–2452. 1976299 10.1128/aem.56.8.2441-2452.1990 PMC184747

[25] James MJ, Lasker BA, McNeil MM, Shelton M, Warnock DW, et al (2000) Use of a repetitive DNA probe to type clinical and environmental isolates of *Aspergillus flavus* from a cluster of cutaneous infections in a neonatal intensive care unit. J Clin Microbiol 38: 3612–3618. 11015372 10.1128/jcm.38.10.3612-3618.2000 PMC87445

[26] Bagyalakshmi R, Therese KL, Madhavan HN (2007) Nucleotide polymorphisms associated with Internal Transcribed Spacer (ITS) regions of ocular isolates of *Aspergillus flavus* . J Microbiol Methods 68: 1–10. 16959342 10.1016/j.mimet.2006.05.021

[27] Heinemann S, Symoens F, Gordts B, Jannes H, Nolard N (2004) Environmental investigations and molecular typing of *Aspergillus flavus* during an outbreak of postoperative infections. J Hosp Infect 57: 149–155. 15183246 10.1016/j.jhin.2004.02.007

[28] Midorikawa GE, Pinheiro MR, Vidigal BS, Arruda MC, Costa FF, et al (2008) Characterization of *Aspergillus flavus* strains from Brazilian Brazil nuts and cashew by RAPD and ribosomal DNA analysis. Lett Appl Microbiol 47: 12–18. 18498318 10.1111/j.1472-765X.2008.02377.x

[29] Montiel D, Dickinson MJ, Lee HA, Dyer PS, Jeenes DJ, et al (2003) Genetic differentiation of the *Aspergillus* section Flavi complex using AFLP fingerprints. Mycol Res 107: 1427–1434. 15000243 10.1017/s0953756203008797

[30] Hadrich I, Makni F, Ayadi A, Ranque S (2010) Microsatellite typing to trace *Aspergillus flavus* infections in a hematology unit. J Clin Microbiol 48: 2396–2401. 20410353 10.1128/JCM.01269-09 PMC2897480

[31] Grubisha LC, Cotty PJ (2010) Genetic isolation among sympatric vegetative compatibility groups of the aflatoxin-producing fungus *Aspergillus flavus* . Mol Ecol 19: 269–280. 20025654 10.1111/j.1365-294X.2009.04467.x

[32] Rudramurthy SM, de Valk HA, Chakrabarti A, Meis JFGM, Klaassen CHW (2011) High Resolution genotyping of clinical *Aspergillus flavus* isolates from India using microsatellites. PLoS One 6: e16086 doi:10.1371/journal.pone.0016086. 21264229 10.1371/journal.pone.0016086 PMC3022034

[33] Grissa I, Bouchon P, Pourcel C, Vergnaud G (2008) On-line resources for bacterial micro-evolution studies using MLVA or CRISPR typing. Biochim 90: 660–668. 10.1016/j.biochi.2007.07.014 17822824

